# Three-Dimensional Microfluidic Tri-Culture Model of the Bone Marrow Microenvironment for Study of Acute Lymphoblastic Leukemia

**DOI:** 10.1371/journal.pone.0140506

**Published:** 2015-10-21

**Authors:** Allison Bruce, Rebecca Evans, Ryan Mezan, Lin Shi, Blake S. Moses, Karen H. Martin, Laura F. Gibson, Yong Yang

**Affiliations:** 1 Department of Chemical Engineering, West Virginia University, Morgantown, West Virginia, United States of America; 2 Alexander B. Osborn Hematopoietic Malignancy and Transplantation Program, Mary Babb Randolph Cancer Center, West Virginia University, Morgantown, West Virginia, United States of America; 3 Department of Neurobiology and Anatomy, West Virginia University, Morgantown, West Virginia, United States of America; 4 Department of Microbiology, Immunology and Cell Biology, West Virginia University, Morgantown, West Virginia, United States of America; Michigan Technological University, UNITED STATES

## Abstract

Acute lymphoblastic leukemia (ALL) initiates and progresses in the bone marrow, and as such, the marrow microenvironment is a critical regulatory component in development of this cancer. However, ALL studies were conducted mainly on flat plastic substrates, which do not recapitulate the characteristics of marrow microenvironments. To study ALL in a model of *in vivo* relevance, we have engineered a 3-D microfluidic cell culture platform. Biologically relevant populations of primary human bone marrow stromal cells, osteoblasts and human leukemic cells representative of an aggressive phenotype were encapsulated in 3-D collagen matrix as the minimal constituents and cultured in a microfluidic platform. The matrix stiffness and fluidic shear stress were controlled in a physiological range. The 3-D microfluidic as well as 3-D static models demonstrated coordinated cell-cell interactions between these cell types compared to the compaction of the 2-D static model. Tumor cell viability in response to an antimetabolite chemotherapeutic agent, cytarabine in tumor cells alone and tri-culture models for 2-D static, 3-D static and 3-D microfluidic models were compared. The present study showed decreased chemotherapeutic drug sensitivity of leukemic cells in 3-D tri-culture models from the 2-D models. The results indicate that the bone marrow microenvironment plays a protective role in tumor cell survival during drug treatment. The engineered 3-D microfluidic tri-culture model enables systematic investigation of effects of cell-cell and cell-matrix interactions on cancer progression and therapeutic intervention in a controllable manner, thus improving our limited comprehension of the role of microenvironmental signals in cancer biology.

## Introduction

Acute lymphoblastic leukemia (ALL), a cancer that starts from overproduction of cancerous, immature white blood cells (lymphoblasts) in bone marrow and spreads to other organs rapidly, affects both children and adults. Approximately 6,000 new ALL cases are diagnosed annually in the US [1]. Although the survival rate of childhood ALL is approaching 90%, the cure rates in adults and subgroups of children with high-risk leukemia are low [2]. The continued progress in development of effective treatment lies in a better understanding of the pathobiology of ALL and the basis of resistance to chemotherapy [3].

ALL initiates and progresses in the bone marrow, and as such, the bone marrow microenvironment is a critical regulatory component in development of this cancer. Bone marrow provides the most common site of leukemia relapse, indicating that this unique anatomical niche is conducive to ALL cell survival *in vivo* [4,5]. It is also a site of metastasis for many solid tumors including breast, lung, and prostate cancer [6–8]. Held in common to all tumor cells that either originate from or migrate to this site is the propensity to be refractory to treatment, thus positioning them to contribute to relapse of disease. Therefore, it is important to model this site appropriately to investigate tumor cell survival in this context and to develop drug screens that incorporate its complexity.

The complexity of the bone marrow microenvironment is significant in terms of cellular constituents and extracellular matrix (ECM). The heterogeneous cell population can be divided into hematopoietic cells and stromal cells including fibroblasts, adipocytes, macrophages, and osteoblasts [5]. The ECM, formed mainly by collagens, glycoproteins such as fibronectin and laminin, and proteoglycans such as heparin sulfate, not only provides the structural scaffold for the cells, but also represents a reservoir of cytokines, chemokines, and growth factors [9]. Various collagens comprise a significant component of the ECM [9] with collagen type I being particularly abundant in the marrow space [10]. Of additional influence on hematopoietic cell development is the stiffness of the matrix, which has profound effects on tumorogenesis [11,12]. Moreover, the interstitial fluid flow in bone, being extremely slow (between 0.1 and 4.0 μm/s [13]), plays an important role in nutrient transport, matrix remodeling and establishment of the microenvironment [14,15]. The interstitial flow has been reported to regulate tumor cell growth, differentiation, migration and metastasis [16–18], and to promote angiogenesis and tumorigenic activity of stromal cells [19]. Collectively, the bone marrow microenvironment contains a complex set of cellular, structural, chemical and mechanical cues necessary to maintain the hematopoietic system.

Conventional cell culture methods using two-dimensional (2-D), stiff plastic surfaces lack characteristics of *in vivo* microenvironment, leading to losses of critical *in vivo* cell phenotype and responsiveness. With recognition of the importance of architecture to the unique anatomy of the bone marrow, effort is warranted to improve on the models to move closer to biological relevance. Three-dimensional (3-D) models have been shown to restore cellular morphology and phenotype characteristics of *in vivo* tumor development [20–23]. Simply switching culture dimensionality from 2-D to 3-D drastically affects cell morphology [24], proliferation [25], differentiation [26], gene and protein expression [21,27–29], and metabolism [30]. Reflecting the impact of dimensionality, GB1 glioma cells were shown to elongate and flatten in 2-D culture, destroying the typical pseudo-spherical morphology and filopodial characteristics, but closely resemble the original *in vivo* phenotype in 3-D culture [24]. Just as cancer cell gene expression patterns can differ, chemotherapy drugs display distinct sensitivities in 2-D versus 3-D environments [21,31,32]. Two-dimensional glioblastoma models were more sensitive to the chemotherapy agent temozolomide than 3-D models or the clinical population [24]. Moreover, acute myeloid leukemia (AML) cells co-cultured with human bone marrow stromal cells (BMSCs) in both 2-D and 3-D scaffolds showed less cytotoxicity of chemotherapy using doxorubicin or cytarabine when compared to the 2-D monoculture condition and the 3-D co-culture achieved the highest resistance to chemotherapy [31].

With the advent of microfluidic technology, the 3-D culture has been performed under dynamic conditions, thus enabling systematic investigation of both physiological and pathological phenomena *in vitro* [33] such as tumor cell migration [34–36], tumor-stromal cell interactions [37], and tumor-endothelial cell interactions [36,38]. For example, breast cancer cells (MDA-MB-231) preferentially migrated along the streamline in a 3-D microfluidic platform, but the direction depended on cell density, chemokine concentration and interstitial flow velocity [39]. Additionally, extravasation and migration distance of MDA-MB-231 was significantly enhanced in a vascularized, osteoblasts-conditioned microenvironment with human BMSC and endothelial cells [40]. These findings emphasize the value of developing 3-D microfluidic models to interrogate biological questions, including those related to the bone marrow microenvironment, as the focus of the current work.

While attempts have been made to develop models with the characteristics of bone marrow microenvironment, ALL studies were conducted mainly on flat plastic substrates, which do not recapitulate the key cellular, structural and mechanical characteristics of the microenvironment. To study ALL in a model of *in vivo* relevance, we have developed a 3-D microfluidic tri-culture model in a biomimetic manner. Biologically relevant populations of primary human BMSCs, osteoblasts and an aggressive form of ALL cells that constitutively expresses the Bcr-Abl fusion protein as the minimal constituents were laden in collagen gels, and cultured in a microfluidic platform. The 3-D microfluidic model was studied with traditional 2-D and 3-D static culture for comparison of tumor cell response to a commonly used chemotherapeutic drug cytarabine (Ara-C).

## Materials and Methods

### Fabrication of a Microfluidic Platform

The microchannels were fabricated via conventional photolithography [41]. The micropattern created with AutoCAD was printed on a transparency film that served as the photomask and SU-8 2050 (MicroChem Corp., Newton, MA, USA) was used as the substrate. A mixture of polydimethylsiloxane (PDMS) resin and curing agent (Sylgard 184 kit, Dow Corning, MI, USA) in a 10:1.05 w/w ratio was poured over the SU-8 mold [42]. After curing at 70°C for 2 hr, the inverse PDMS layer with microchannels was peeled from the SU-8 mold.

The PDMS layer was assembled onto a glass coverslip to form a microfluidic platform by using either conventional oxygen plasma assembly or novel microtransfer assembly (μTA) method as reported previously [43]. Oxygen plasma provides a permanent assembly while μTA is reversible. In oxygen plasma assembly, the PDMS layer with microchannels and the glass coverslip were exposed to oxygen plasma at 300 mTorr (40 Pa), 50 watt for 15 s in a March PX-250 Plasma Asher (Nordson Co., Westlake, OH, USA), brought into contact and then placed in an oven at 125°C for 15 min to complete the assembly. In μTA, 5 wt.% PDMS prepolymer in hexane solution was spin-coated onto a Si wafer to form a thin film, which served as an adhesive layer to assemble PDMS microchannels onto a glass coverslip. The assembly was then post-cured at 80°C for 2 hr. The bond strength of the μTA assembly is controlled by pre-baking of the adhesive layer, thus allowing to peel the PDMS layer off from the glass coverslip after cell culture and to retrieve the cell-laden hydrogel matrix for analysis.

The microfluidic platform was sterilized by injecting and retaining 70% ethanol within the microchannels for 30 min, and then by UV exposure for 30 min, followed by phosphate buffered saline (PBS; Mediatech, Manassas, VA, USA) wash prior to cell loading.

### Cell Culture

The human Philadelphia chromosome positive (Ph+) B lineage ALL cell line SUP-B15 (CRL-1929; ATCC, Manassas, VA, USA) was maintained in Iscove's DMEM (Mediatech, Manassas, VA, USA) supplemented with 10% fetal bovine serum (FBS; Hyclone, Logan, UT, USA), 2 mM L-glutamine (Life Technologies, Carlsbad, CA, USA), 0.05 μM 2-β−mercaptoethanol (Sigma-Aldrich, St. Louis, MO, USA) and 1% penicillin/streptomycin (Sigma-Aldrich). Human bone marrow stromal cells (BMSC) were from the cytogenetics lab at West Virginia University and were considered fully and completely de-identified stromal pathology waste specimens. The de-identified pathology waste specimens were not consented, and exempt from the West Virginia University Institutional Review Board review. Primary BMSC were maintained in α-modification of Eagle's medium (α-MEM; Life Technologies) supplemented with 10% FBS, 1% L-glutamine, 1% penicillin/streptomycin, and 0.1% 2-β−mercaptoethanol. Human osteoblasts (HOB) were purchased from Promocell (Heidelberg, Germany) and maintained in Osteoblast Growth Medium (C-2700, PromoCell). For tri-cultures of SUP-B15, BMSC and HOB, the complete culture medium for SUP-B15 was used.

### Tumor Models

SUP-B15 leukemic cells monoculture and tri-culture with BMSC and HOB were performed in 2-D static, 3-D static and 3-D microfluidic models. The total cell density was maintained at 10 x 10^6^ cells/mL. In tri-culture experiments, SUP-B15 cells were mixed with BMSC and HOB at a 10:2:2 ratio.

In the 3-D microfluidic model, the cells were resuspended in 100 μL complete culture medium, gently mixed with 10% 10X PBS, 2% NaOH (Sigma-Aldrich) and 88% collagen I (rat-tail; BD Biosciences, Franklin Lakes, NJ, USA), and then loaded into the microfluidic platform. The platform was incubated at 37°C for 4 hr to allow collagen I to gelate before the culture media was continuously pumped through the platform at a flow rate of 300 μL per day using a Harvard Apparatus PhD Ultra Pump (Harvard Apparatus, Holliston, MA, USA) to sustain cell culture for a predefined period of time. Similarly, in the 3-D static model, the cell-laden collagen I was loaded into the microchannels and the culture medium was replaced every 2 days. In the 2-D static model, BMSC or BMSC + HOB were seeded onto glass coverslips in 24-well cell culture dishes to provide an 80–90% confluent monolayer. Twenty four hours later, SUP-B15 cells were seeded onto the monolayer.

### Cell Labeling and Immunofluorescence Staining

To examine cell-cell interactions in the tri-culture models, SUP-B15 cells, BMSC and HOB were fluorescently labeled using CellTracker Green CMFDA, CellTracker Red CMTPX and CellTrace Far Red DDAO-SE, respectively (Life Technologies).

To examine cell-matrix interactions, after the pre-determined culture period (4 or 7 days), the cells were fixed in 4% paraformaldehyde (PFA, Sigma-Aldrich) for 30 min, blocked and permeabilized with 0.5% Triton X-100 in PBS and 7 mg/mL gelatin from fish skin (G7765, Sigma-Aldrich) for 30 min, stained with Ki67 rabbit monoclonal antibody (1:200; Cell Signaling, Danvers, MA, USA), followed by the Alexa Fluor 488 goat anti-rabbit secondary antibody (1:200; Life Technologies). F-actin was stained with TRITC-phalloidin (Sigma-Aldrich), and the nuclei were stained and mounted using ProLong Gold Antifade Reagent with 4,6-diamidino-2-phenylindole (DAPI, Life Technologies).

Imaging was performed with a Zeiss LSM 510 upright confocal microscope. For 3-D visualization, confocal z-stack imaging with 1 μm intervals between planar images was conducted.

### Chemotherapeutic Treatment

Antimetabolite chemotherapeutic drug, Ara-C (Sigma-Aldrich) was used for the chemoresistance study. Ara-C was stored at -80°C at 10 mg/mL, and diluted in the complete culture medium used for leukemic cell culture immediately before use. After 72 h incubation, tumor cells cultured in 2-D and 3-D under static conditions were treated with 1 μM Ara-C for 48 h to best mimic an ALL patient’s drug serum concentration during treatment, while 3-D microfluidic platforms were exposed to continuous flow of 1 μM Ara-C in complete culture medium for 48 h. Viability was evaluated by both flow cytometry and immunofluorescence image analysis.

### Viability Assays

For flow cytometry analysis, collection of tumor cells from the 3-D models was accomplished by incubation of cell-laden matrices in type I collagenase (Life Technologies). The collagenase was used to digest collagen I according to the manufacturer’s protocol. Apoptotic cells were detected by staining with Annexin V (Alexa Fluor 555 conjugate; Life Technologies) and gated against cells stained with CellTracker Green CMFDA. Data were acquired using a FACSFortessa flow cytometer with Cell Quest Pro software (BD Biosciences).

Cell viability was also assessed by immunofluorescence staining. Apoptotic cells were detected by staining with Alexa Fluor 555 Annexin V at 4°C overnight. Samples were washed, fixed with 4% PFA, and imaged on the same day. Experiments were repeated in triplicate.

### Confocal Image Analysis

Confocal images were analyzed using Imaris software (Bitplane, South Windsor, CT, USA). To reconstruct the 3-D model, the surface function was used to create representative objects in the FITC (green), TRITC (red), and DAPI (blue) channels. The threshold for each individual channel was adjusted to a level where the superimposed confocal image matched that of the created objects. Once the objects were created, the image could then be rotated for different angle views. Determining cell viability followed a similar protocol. The objects were created only in FITC and TRITC channels. The resulting objects were then filtered based on size to remove unwanted noise and to evaluate only tumor cell viability. Remaining objects were counted based on color. Red Annexin V-positive cells were counted as not viable, while green Annexin V-negative cells were counted as viable.

### Statistical Analysis

Viability percentages were expressed as mean ± standard deviation from three independent experiments. Immunofluorescence-stained images of 200 tumor cells per sample were evaluated. The differences between groups were analyzed by one-way ANOVA and Tukey’s method for multiple comparisons at confidence interval levels of 95%.

## Results and Discussion

### 3-D Microfluidic Tri-Culture Model

**Fig 1:**
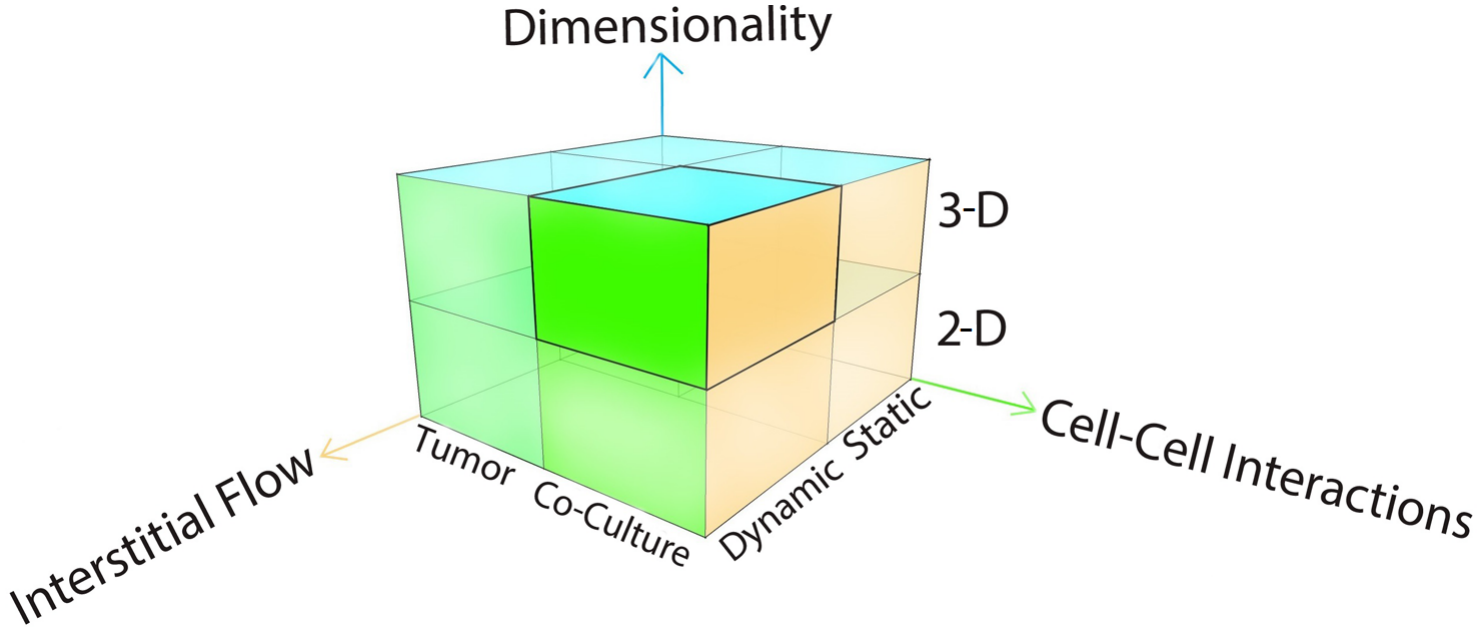
Design considerations of a tumor model.

The current study sought to develop a 3-D microfluidic model with utility for investigation of tumor cell biology in the context of the bone marrow microenvironment as the site of initiation of leukemic disease and as the frequent site of metastatic malignancies characterized by therapeutic resistance. Inherent to this design were considerations of dimensionality, cell-cell interactions and interstitial flow (Fig 1) as critical parameters that impact on cell morphology, signaling and therapeutic resistance.

**Fig 2:**
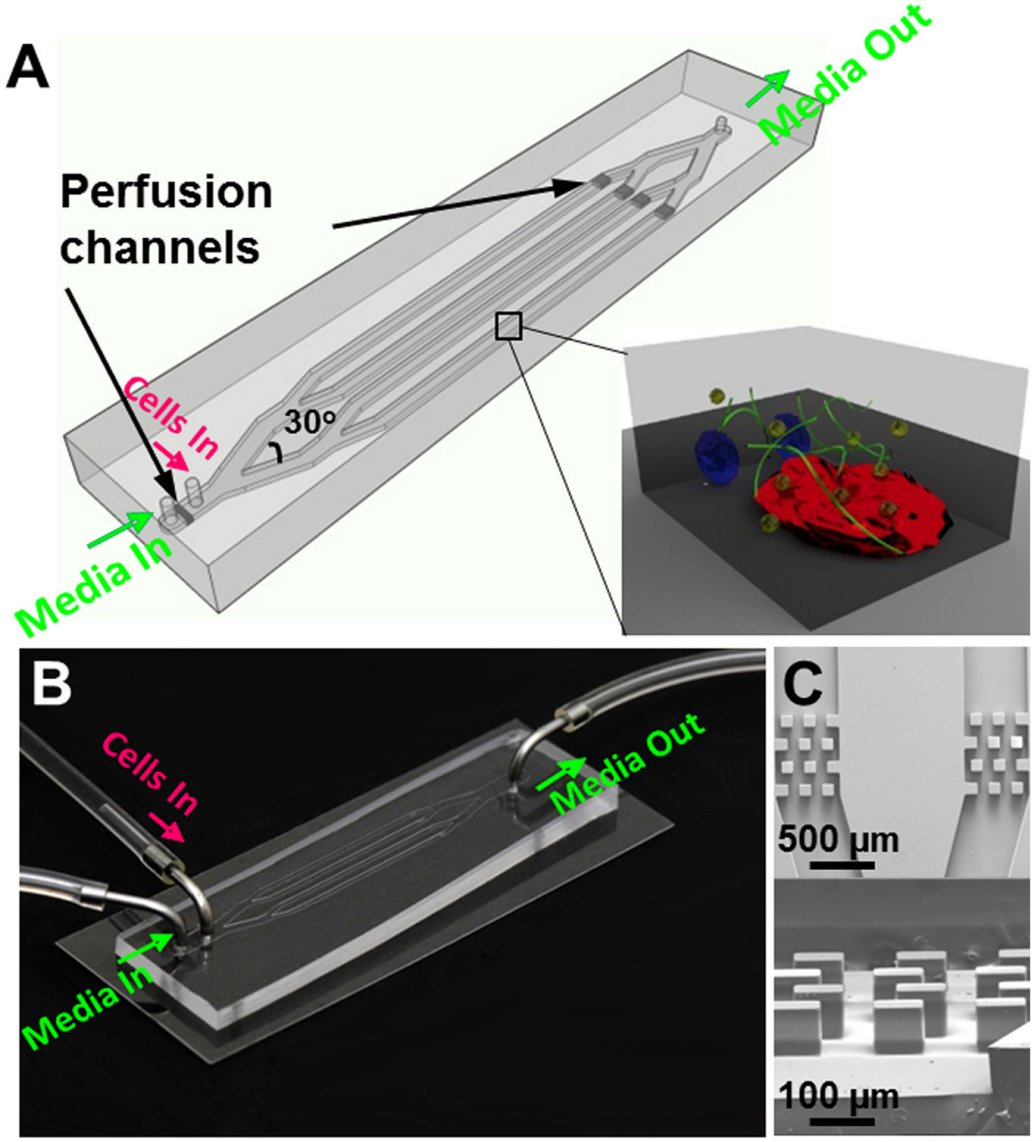
A typical 3-D microfluidic tri-culture model. (A) The microfluidic platform consists of 4 microchannels, each 500 μm in width, 75 μm in height and 2 cm in length. The divergent angles are 30°. Near the inlets and outlet there are perfusion channels. The enlarged boxed area illustrates multiple cell types embedded in a 3-D matrix. (B) Optical image of a microfluidic platform. (C) SEM images of top view (upper panel) and side view (lower panel) of the perfusion channels.

A typical 3-D microfluidic model was shown in Fig 2. Tumor cells (green), BMSC (red) and HOB (blue) embedded in collagen I were injected through the “Cells In” inlet into four microchannels, which were 500 μm in width, 75 μm in height and 2 cm in length (Fig 2A and 2B). After collagen I gelated, the culture medium was pumped through a “Media In” inlet into the four microchannels for a predefined period of time. Near the inlet and outlet, perfusion channels comprising of four rows of 100 μm x 100 μm columns with a spacing of 80 μm (Fig 2C) were fabricated to help retain the cells within the main microchannels (S1 Fig). The determination of the microchannel dimensions considered the nutrient diffusion limits and provided the space for tumor cells to interact with other cells and matrix [44].

In addition to a generic 3-D environment, the specific leukemia microenvironment needs to be represented by its cellular components. BMSC [45] and osteoblasts [46,47] comprise just two of the well characterized populations that are essential to the developmental support of both normal and leukemic cells in postnatal bone marrow, and thereby were included as representative, but not exclusive, elements of the bone marrow microenvironment.

Collagen I was chosen for inclusion for several reasons. Although collagen types I and IV, fibronectin, laminin, and heparin sulfate have been identified as important components of the bone marrow ECM [10], the relative composition of the ECM is self-regulating and yet unquantified. Indeed, collagen I has been shown to be predominant in the bone marrow ECM [9]. Moreover, many tumors, such as breast, pancreatic, squamous cell carcinoma, expressed significantly higher collagen I in the ECM *in vivo* than the normal tissues [48–50]. Additionally, collagen stiffness has been shown to positively correlate with mammary tumor growth and metastasis [51,52]. In the current study, we mainly used a concentration of collagen I at 2 mg/mL, corresponding to 300 Pa in stiffness [53], which agreed with the macroscopic measurements of extracted bone marrow, <300 Pa [54]. Collagen I at 4 mg/mL (1200 Pa in stiffness [53]) was also used to demonstrate the matrix stiffness effect.

The velocity profile within the microchannels deviated from Poiseuille flow because collagen hydrogel is compliant and porous of various pore size and cells may induce matrix remodeling [19]. By tracking the movement of injected FluoSpheres microspheres (F-13082, 1 μm, orange 540/560 nm; Life Technologies), the velocity was determined to be 0.27 ± 0.18 μm/s (S2 Fig). As such, in the current experiments flow was approximated to be that which has been reported in the interstitial space of the marrow [13].

### Cell-Cell Interactions

**Fig 3:**
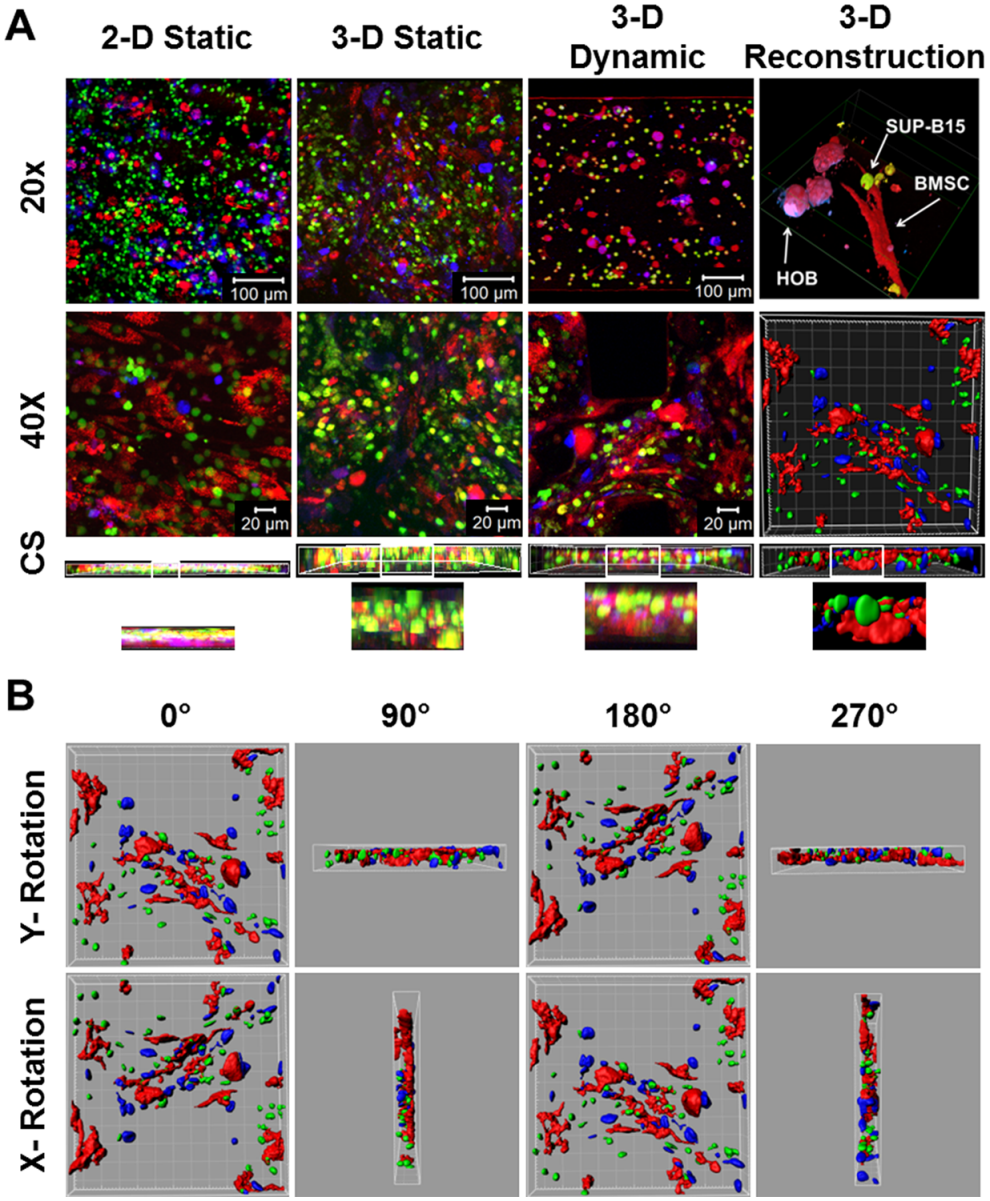
Cell-cell interactions in the tumor models. (A) Confocal and Imaris reconstruction images were presented of the tri-culture of SUP-B15 tumor cells (green), BMSC (red) and HOB (blue) across the tumor models at low magnification (20X), high magnification (40X) and high magnification cross sections (CS) of 40X images. The cell-cell interactions in z-direction in the boxed region of the CS images were enlarged in Row 4, where the heights for 2-D and 3-D CS images were 10 μm and 30 μm, respectively. (B) 3-D reconstruction images rotated via x- and y-axis revealing cell-cell interactions.

Confocal and reconstruction images (Fig 3) show that in both 2-D and 3-D static as well as in 3-D dynamic experimental circumstances, all cell populations can be readily discerned with 3-D reconstruction demonstrating direct cell-cell interactions with BMSC and HOB. Cross sections (CS) (Fig 3A) demonstrate the compaction of the 2-D static model compared to the 3-D circumstance in which the dimensionality can be appreciated. The cell-cell interactions are further emphasized upon rotation of the 3-D reconstruction images around the X and Y axis indicating tumor cell interaction with structural cells (BMSC and HOB) in three dimensions (Fig 3B and S1 Video).

The 2-D static model results in a non-physiological environment without any discernable niches. Cells within a 3-D environment behave very differently from cells on 2-D substrates, as cell—matrix interactions affect tumor cell migration and invasion, trigger signaling pathways and cellular responses in 3-D, which may not be observed in 2-D [23]. The 3-D static model does create a microenvironment, but the absence of dynamic stresses causes the cells to diffuse and lack interaction.

### Cell-Matrix Interactions

**Fig 4:**
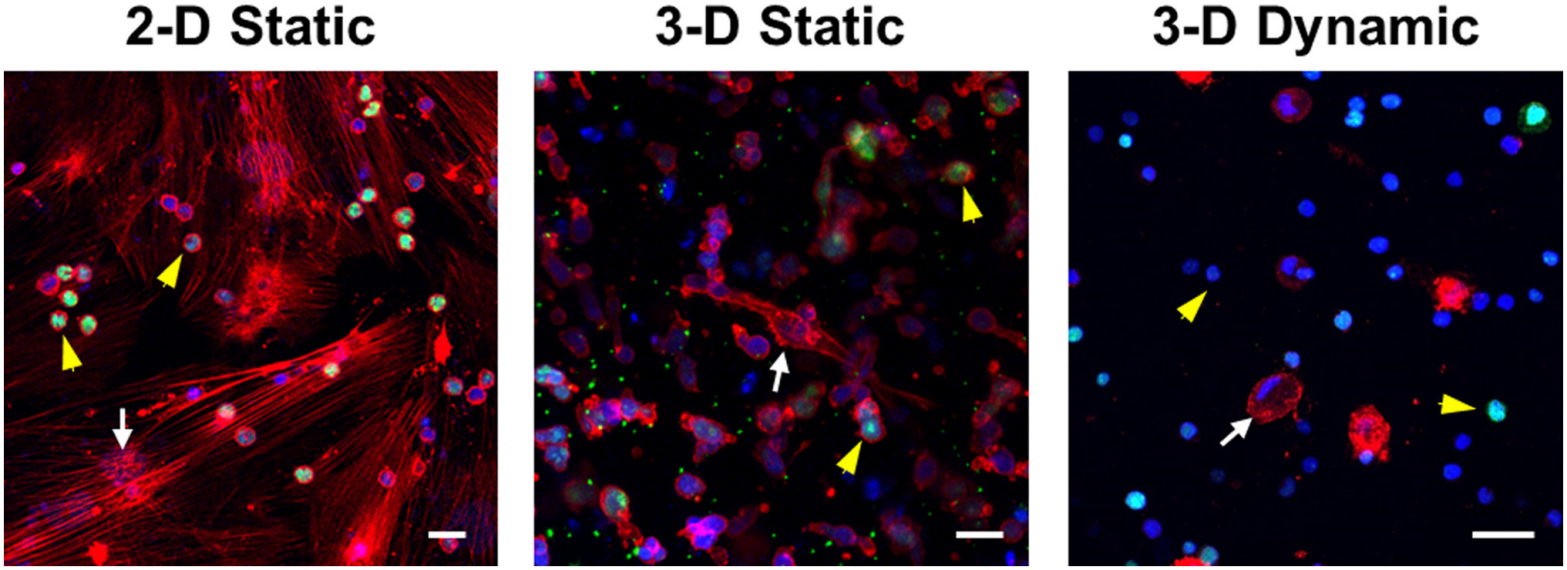
Confocal images of SUP-B15 and BMSC co-cultured in 2-D static, 3-D static and 3-D dynamic models. The yellow arrowheads and white arrows point to SUP-B15 cells and BMSC, respectively. The actin filaments were stained with phalloidin in red, the nuclei were stained with DAPI in blue, and the nuclei of proliferating cells were stained with Ki67 in green. Scale bars: 20 μm.

Of interest in the application of this model is the capacity for evaluating effects of cell-matrix interactions on tumor progression and therapeutic resistance. In order to examine the effects of cell-matrix interaction, the concentration of collagen was reduced, and tumor cell proliferation was examined after four days in culture. Ki67 immunostaining (Fig 4) indicated that tumor cells were proliferating, but no differences were noted in the quantitation between the three tumor models (data not shown). It also demonstrates that the 3-D microfluidic platform and fixation of cells at the cessation of culture are both permissive for detection of proteins of interest by fluorescent microscopy. The structural BMSC, however, displayed distinguishable morphologies among these three models as well as compared to the models with higher concentrations. As shown in Fig 4, BMSC substantially elongated with distinct stress fibers in the 2-D static model while BMSC spreading was reduced in the 3-D static conditions and became limited in the 3-D dynamic model. No significant BMSC spreading was observed up to 2 weeks culture in 3-D dynamic model. The cell spreading in the 3-D models was much less than that was demonstrated in Fig 3, where BMSC spreading was significant even in 3-D dynamic model. The difference can be attributed to the matrix stiffness alteration. The concentration of collagen I used in Fig 3 was 4 mg/mL and the corresponding stiffness was estimated to be 1200 Pa [53], three-fold stiffer than collagen I matrix in Fig 4 (300 Pa for 2 mg/mL). Decrease in collagen cross-linking and matrix stiffness reduced cell spreading and progression [52]. Moreover, the interstitial flow is expected to remodel the matrix by facilitating collagen degradation through removal of degraded products. There is a reason to believe that the flow-induced remodeling lowered the matrix stiffness and thus limited BMSC spreading. It has been shown that substrate stiffness can influence cytotoxic response and drug resistance [55]. The 3-D microfluidic model, thereby, creates a more accurate model of the niche, with shear forces allowing the different cells in tri-culture to interact in a dynamic condition and form a more authentic microenvironmental niche.

### Therapeutic Resistance

Beyond ability to examine cell-cell and cell-matrix interactions following culture, we compared chemoresistance of tumor cells to Ara-C, a chemotherapy agent used mainly in the treatment of cancers of white blood cells such as AML and non-Hodgkin lymphoma.

**Fig 5:**
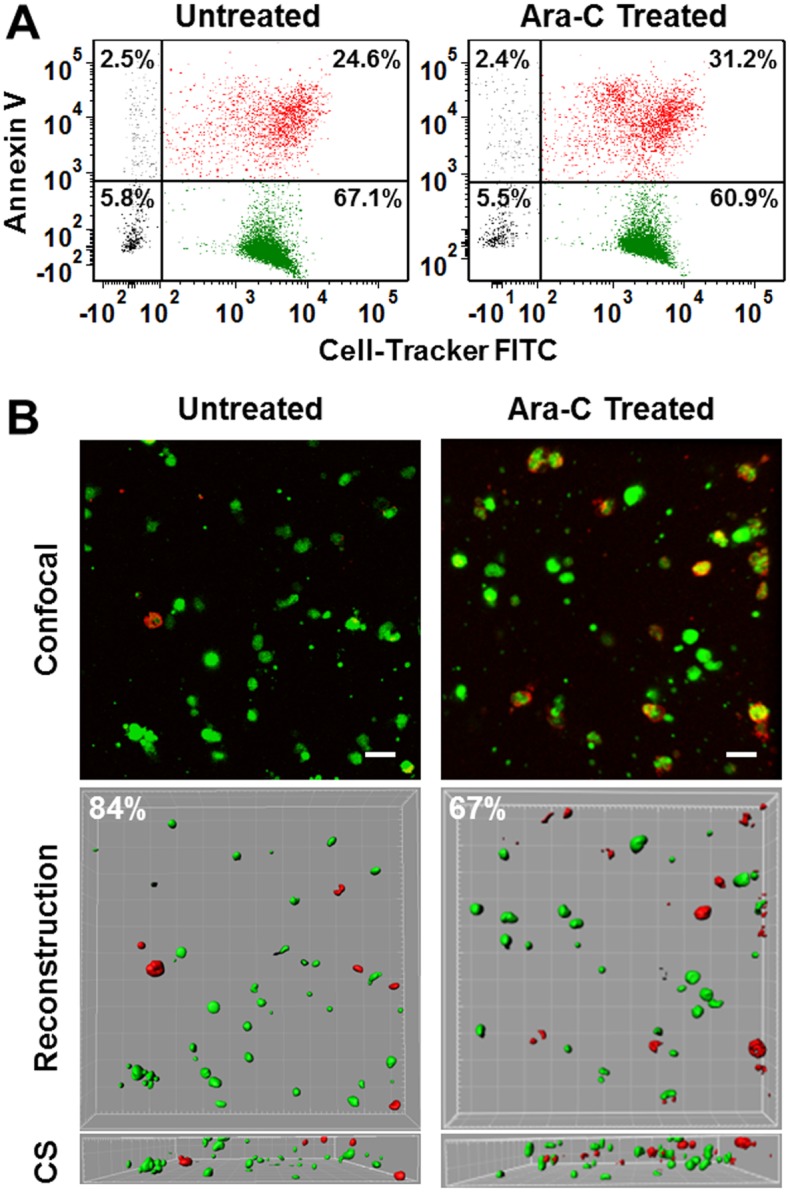
Viability analysis using Annexin V. (A) Representative flow cytometry analysis for untreated (left) and Ara-C treated (right) 3-D static tri-culture models. (B) Representative immunofluorescence image analysis of untreated (left) and treated (right) 3-D static tri-culture models. The immunofluorescence images are the projection of z-stack confocal images. Confocal images were reconstructed using Imaris and also presented from a cross-sectional (CS) view. Scale bars: 20 μm.

As demonstrated in Fig 5A, flow cytometry analysis showed an increase in apoptotic Annexin-positive cells (red, upper right) and a corresponding decrease in viable cells (green, lower right) when a tumor cell population was treated with Ara-C. The results agree with confocal microscopy. Ara-C treatment resulted in more apoptotic cells (red) and a corresponding decrease in viable cells (green) (Fig 5B). Viability was also enumerated via the less sensitive method of trypan blue exclusion, which displayed similar trend to the immunofluorescence image analysis of Annexin V staining. Only immunofluorescence image analysis results were discussed here.

**Fig 6:**
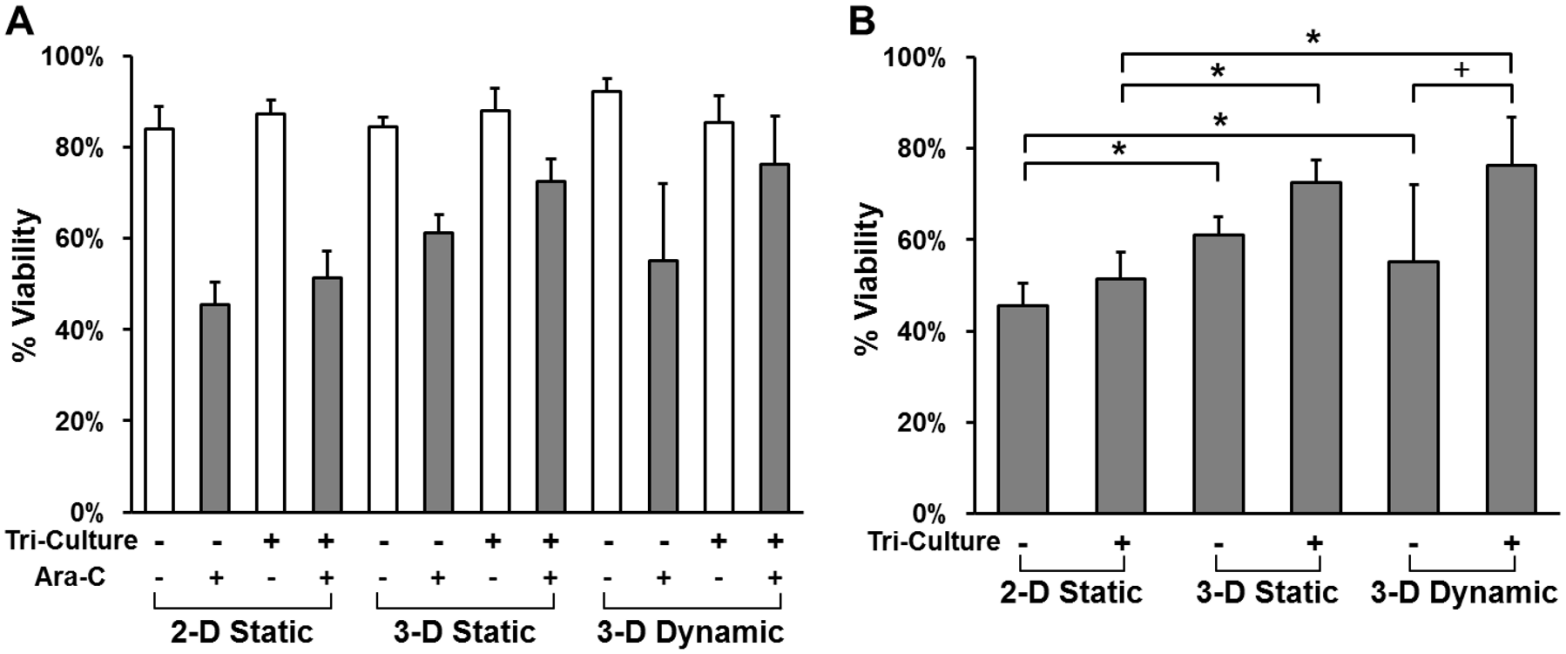
Comparison in chemoresistance of tumor cells to Ara-C among a variety of tumor models. (A) The effects of dimensionality, tri-culture and interstitial flow on the viability of tumor cells. (B) The effects of 3-D culture on tumor cell viability under the tri-culture condition. Significant difference between two groups is indicated by + where *p* < 0.1, * where *p* < 0.05.

The chemoresistance results were summarized in Fig 6A. As highlighted in Fig 6B, tri-culture with stromal cells and osteoblasts enhanced leukemic cell survival during chemotherapy exposure in both 2-D and 3-D models regardless of whether the 3-D configuration was static or had a steady media flow during culture. It has been reported that both stromal cells and osteoblasts enhanced leukemic cell survival during chemotherapy exposure with previous experiments typically completed in traditional 2-D cultures [56,57]. Note that in 3-D dynamic models the continuous flow of Ara-C was applied. With a higher drug dosage than 3-D static models, where drug concentration decayed over time for both tumor monoculture and tri-culture, the tumor cells still maintained the similar viability in 3-D dynamic models, indicating more microenvironment protection in 3-D dynamic models. The relevance of this observation is that surviving tumor cells in the context of leukemia have been shown to directly correlate with the likelihood of relapse of disease and as such more biologically relevant models to interrogate the protective effect of critical elements of the microenvironment are warranted [4,58]. Precise control over the fluidic flow permits the recapitulation of clinically relevant drug dosages to determine parameters for optimal dosage concentrations, combinations and sequences.

The microenvironment protection in 3-D models was more significant than 2-D models for both tumor monoculture and tri-cultures (Fig 6B). Tumor cells cultured in 3-D have been shown to be less sensitive to apoptosis induced by radio-chemo treatments or by death receptor ligation compared to cells cultured in 2-D [59,60]. Cytotoxic effects of chemotherapeutic agents have also been shown to be significantly reduced in cells cultured in 3-D [21,31,32]. The majority of the observed chemoresistance difference can be attributed to differences in cell-matrix interactions in 3-D environment compared with 2-D culture, which were mediated by changes in integrin receptor localization and activation, and intracellular signal transduction [61]. It has been shown that inhibition of integrin signaling in combination with chemotherapy led to an improvement in cytotoxic response [62,63]. In addition, 3-D hydrogels, such as collagen matrices, can act as a barrier [64–66] to counteract drug delivery, ultimately decreasing drug efficacy within the target tissue [67,68].

These data suggest that investigation of leukemic cell protection by bone marrow microenvironment cues in traditional 2-D culture models can provide meaningful information in which trends are confirmed in 3-D culture, but that more accurate measures of the magnitude of effect may require further development of 3-D models. No readouts were observed that were not consistent between 2-D and 3-D based experiments, and as such, practical downstream evaluation of cells may also drive the experimental design choice. Potentially experiments in which large numbers of tumor cells are required for recovery at the cessation of cytotoxic exposure may be best aligned with 2-D approaches, with 3-D lending itself best to single cell analyses or polymerase chain reaction (PCR) based applications. We have documented that leukemic cells can be recovered via collagenase digestion and cell sorting with high quality RNA that is appropriate for PCR (data not shown). This will broaden the realm of experiments that will be possible related to gene expression studies in this microenvironment model.

## Conclusions

Relapse of leukemic disease in high risk categories of ALL remains a significant clinical challenge. In combination with appropriate *in vivo* models it remains essential to optimize *in vitro* models to more accurately recapitulate the complexity of the bone marrow microenvironment to provide new tools for investigation of the mechanisms that underpin survival of residual disease and relapse. The microfluidic model in the current study represents one step in this effort with relevance to both leukemic disease as well as metastasis of solid tumors to the bone marrow where they also benefit from the protective niche during therapy. Our results show decreased chemotherapeutic drug sensitivity of leukemic cells in the 3-D microfluidic model from the 2-D models. The engineered 3-D microfluidic tri-culture model allows precise control over the mechanical properties of matrix and fluidic shear stress and enables systematic investigation of effects of cell-cell and cell-matrix interactions on cancer initiation and progression, thus improving our limited comprehension of the role of microenvironmental signals in cancer biology. It is also recognized that the resulting 3-D models still clearly reflect a “reductionist” approach. Additional consideration as the model progresses to increased translational relevance would be the influence of low oxygen tension along with additional cell types and extracellular matrix components. Future explorations using this biomimetic model is expected to advance our understanding of the pathobiology of ALL and to help identify new therapeutic targets and more effective cancer treatment options.

## Supporting Information

S1 Data Data used for generating Fig 6. (XLSX)

S1 Fig The cells uniformly distributed in the main microchannel. (TIF)

S2 Fig Flow velocity through the interstitial space was calculated by tracking fluorescent microspheres in the chips using *Imaris*. (A) Snapshot of fluorescent microspheres (red) flow through a microchannel. (B) Histogram of velocity distribution.
(TIF)

S1 Video Reconstruction of 3-D microfluidic tri-culture model revealed cell-cell interaction in all directions. (MP4)
