# Correction: Three-Dimensional Microfluidic Tri-Culture Model of the Bone Marrow Microenvironment for Study of Acute Lymphoblastic Leukemia

**DOI:** 10.1371/journal.pone.0146203

**Published:** 2015-12-30

**Authors:** The PLOS ONE Staff

The following information is missing from the Funding section. The publisher apologizes for the error. The authors acknowledge support for A.B. and Y.Y. from the NSF BRIGE grant EEC 1227766 and NSF EPS 1003907, for L.G. in part, from R01 HL056888, R01 CA134573, P30 RR032138, the Alexander B. Osborn Hematopoietic Malignancy and Transplantation Program, and R.E. from the Mary Babb Randolph Cancer Center (MBRCC)'s Hostler-Childs Cancer Research Fund. We also gratefully acknowledge use of the WVU Microscope Imaging Facility, which has been supported by the MBRCC and NIH grants P30 GM103488 and P20 RR016477, the WVU Flow Cytometry Core supported by MBRCC CoBRE grant GM103488/RR032138, Fortessa S10 grant OD016165 and WV InBRE grant GM103434, and the WVU Shared Research Facilities which are supported, in part, by NSF EPS-1003907. The funders had no role in study design, data collection and analysis, decision to publish, or preparation of the manuscript.
