# Supplementary figures and images for: Three-Dimensional Microfluidic Tri-Culture Model of the Bone Marrow Microenvironment for Study of Acute Lymphoblastic Leukemia
Source: PLoS One. 2015 Oct 21;10(10):e0140506. doi: 10.1371/journal.pone.0140506 (PMC4619215; doi:10.1371/journal.pone.0140506)

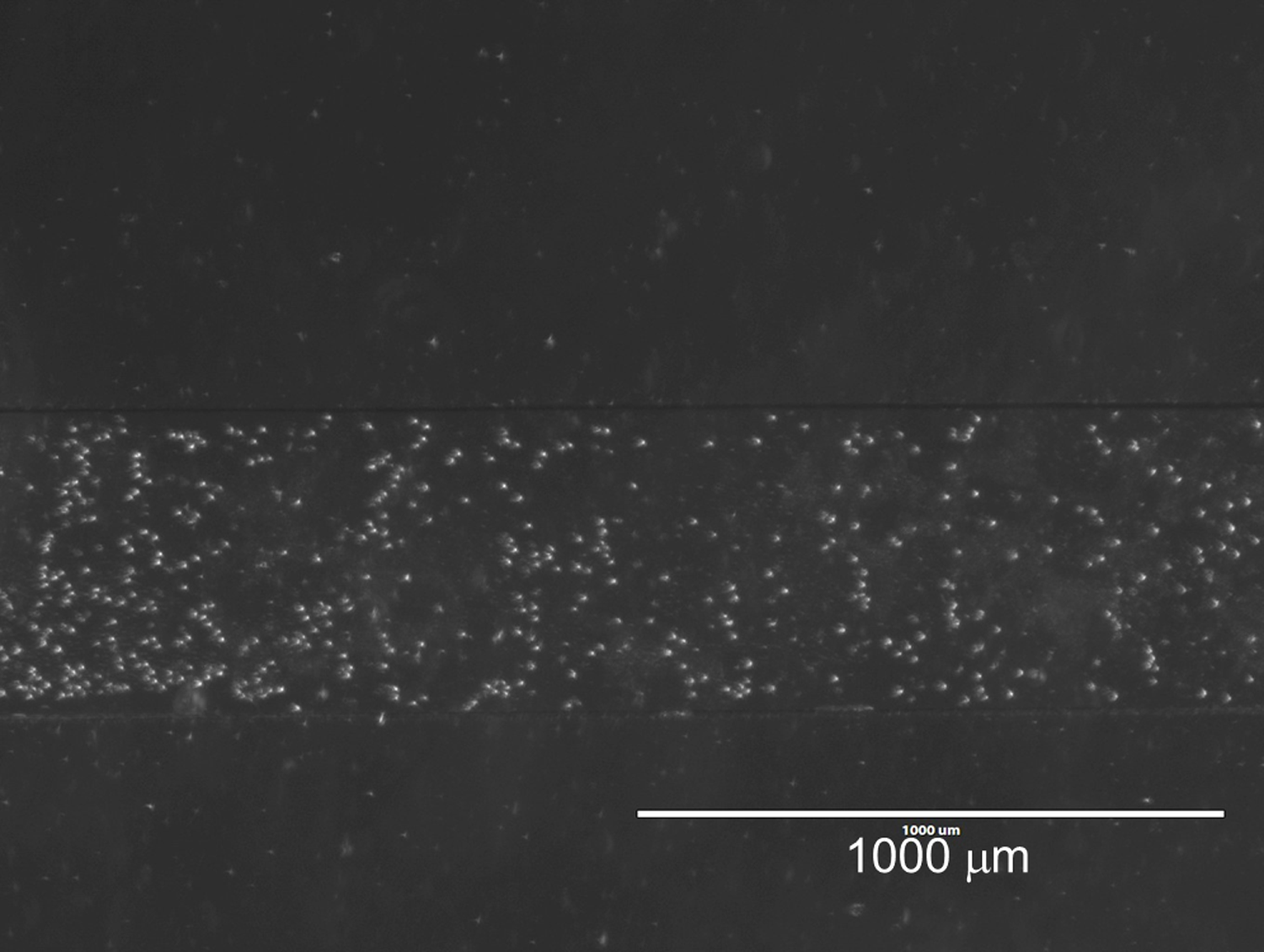

Supplement: S1 Fig — (TIF) [file pone.0140506.s002.tif]

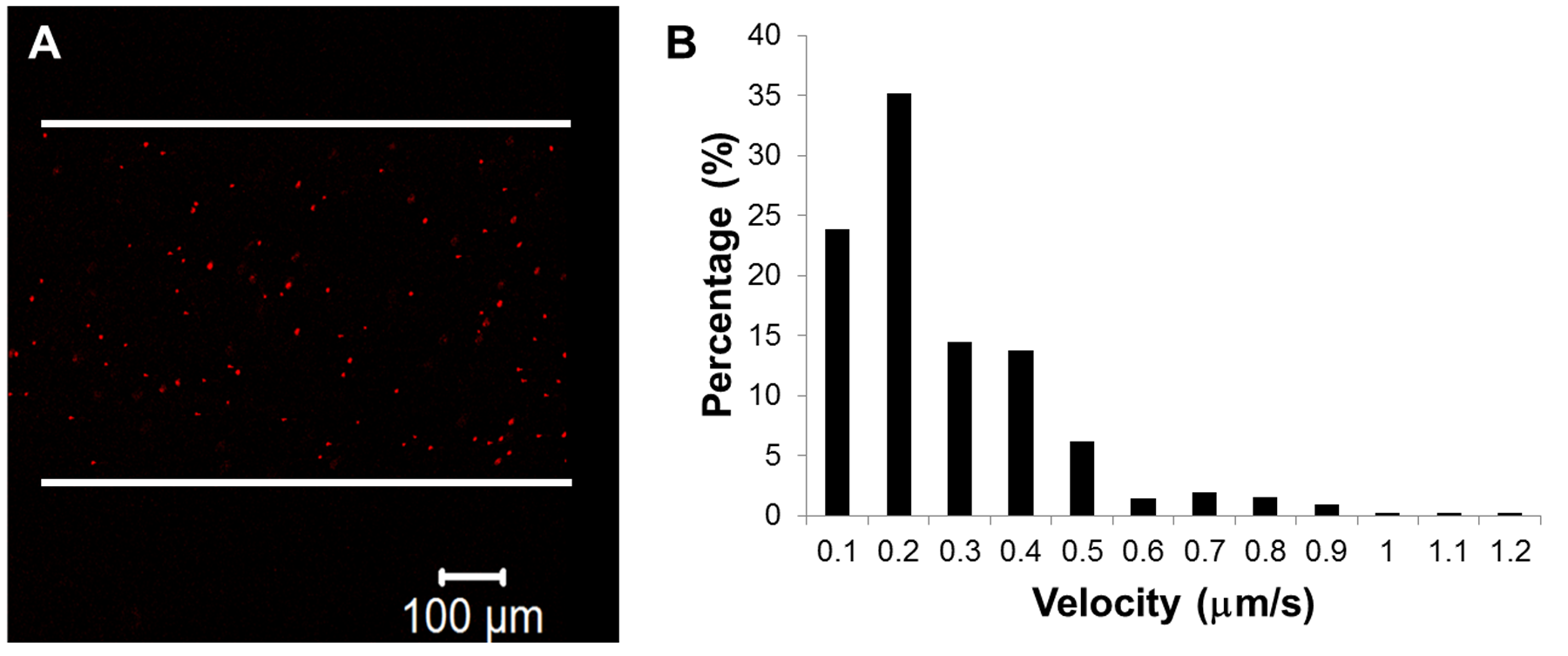

Supplement: S2 Fig — (A) Snapshot of fluorescent microspheres (red) flow through a microchannel. (B) Histogram of velocity distribution. (TIF) [file pone.0140506.s003.tif]
